# Leveraging Electronic Health Dataset to Identify Co‐existing Multimorbidity in Individuals with Alzheimer's Disease in the Midwestern United States

**DOI:** 10.1002/alz70860_100675

**Published:** 2025-12-23

**Authors:** Nai‐Ching Chi, Patrick Lozier, Nick Street, Kathleen Buckwalter, Barbara Rakel, Sue Gardner, Yelena Perkhounkova, Maria Hein, Pui Ying Yew, Chih‐Lin Chi, Nanle Joseph Gusen

**Affiliations:** ^1^ University of Iowa, Iowa City, IA, USA; ^2^ University of Minnesota, Minneapolis, MN, USA

## Abstract

**Background:**

Alzheimer's Disease (AD) frequently co‐occurs with multiple chronic conditions (MCCs). This study aimed to identify specific combinations of MCCs, utilizing detailed ICD‐10 groupings to inform targeted prevention strategies.

**Method:**

We analyzed de‐identified electronic health records (EHR) of 2,629 AD patients from the University of Iowa Hospitals and Clinics (2015‐2021). Patient diagnoses were grouped into their first 3‐level ICD‐10 codes. The Apriori algorithm was employed to identify co‐occurring ICD‐10 groups, iteratively analyzing singular, dyad, triad, and tetrad combinations. Support (frequency) and Leverage (correlation) metrics were used to identify significant patterns.

**Result:**

46 common ICD‐10 groups were identified. Hypertension (I10; 43.3%), lipoprotein metabolism disorders (E78; 31.5%), major depressive disorder (F33; 23.6%), and diabetes (E11) were the most frequent singular co‐occurrences. Hypertension consistently appeared in the most common dyads (e.g., I10‐E78: 21.4%, I10‐F33: 13.1%, I10‐E11: 10.9%), with I10‐E78 exhibiting the highest correlation (7.8% more frequent than expected). Other highly correlated dyads included major depression‐anxiety (F33‐F41; 4.5%) and lipoprotein metabolism‐ischemic heart disease (E78‐I25; 3.9%). These dyads formed the most common/correlated triads (e.g., I10‐E78‐F33: 7.7%/4.5%, I10‐E78‐E11: 6.7%/4.2%, I10‐E78‐I25: 6.6%/4.5%). Notably, triads involving hypertension‐ischemic heart disease (I10‐I25) with gastroesophageal (K21), lipoprotein metabolic (E78), and sleep disorders (G47) demonstrated frequencies more than two‐fold higher than expected (high correlation). The most common and correlated tetrads included hypertension‐lipoprotein metabolism‐major depression (I10‐E78‐F33) with anxiety (F41), gastroesophageal reflux (K21), sleep disorders (G47), and other hypothyroidism (E03), as well as hypertension‐lipoprotein metabolism‐gastroesophageal reflux‐ischemic heart disease (I10‐E78‐K21‐I25).

**Conclusion:**

This study identified highly prevalent and correlated combinations of MCCs in AD patients, including well‐established risk factors. These findings have crucial implications for developing targeted disease prevention strategies in this vulnerable population.

